# Construction of functional tissue-engineered bone using cell sheet technology in a canine model

**DOI:** 10.3892/etm.2014.1514

**Published:** 2014-01-29

**Authors:** TAO CHEN, YANHUI WANG, LINGXUE BU, NINGYI LI

**Affiliations:** Department of Oral and Maxillofacial Surgery, Affiliated Hospital of Qingdao University Medical College, Qingdao, Shandong 266003, P.R. China

**Keywords:** mesenchymal stem cells, cell sheet technology, tissue-engineered bone

## Abstract

The aim of the present study was to construct functional tissue-engineered bone with cell sheet technology and compare the efficacy of this method with that of traditional bone tissue engineering techniques. Canine bone mesenchymal stem cells (BMSCs) were isolated using density gradient centrifugation and then cultured. The BMSCs were induced to differentiate into osteoblasts and cultured in temperature-responsive culture dishes. The BMSCs detached automatically from the temperature-responsive culture dishes when the temperature was reduced to 20°C, forming an intact cell sheet. Demineralized bone matrix (DBM) and platelet-rich plasma (PRP) were prepared and used to construct a DBM/PRP/BMSC cell sheet/BMSC complex, which was implanted under the left latissimus dorsi muscle in a dog model. A DBM/PRP/BMSC complex was used as a control and implanted under the right latissimus dorsi muscle in the dog model. Immunoblot assays were performed to detect the levels of growth factors. Osteogenesis was observed to be induced significantly more effectively in the DBM/PRP/BMSC cell sheet/BMSC implants than in the DBM/PRP/BMSC implants. Immunoblot assay results indicated that the levels of the growth factors platelet-derived growth factor (PDGF) and vascular endothelial growth factor (VEGF) in the experimental group were 3.2- and 2.5-fold higher compared with those in the control group, respectively. These results indicated that the BMSC cell sheets were functional and more effective than the control cell complex. Therefore, cell sheet technology may be used for the effective construction of functional tissue-engineered bone with ideal properties.

## Introduction

Traditional bone tissue engineering technology is widely used in the preparation of tissue-engineered bone. The traditional method extracts inoculated cells for transfer by trypsin digestion, which results in the loss of a large number of cells and a reduction in cell activity. Thus, dense bone tissue is rarely formed by the traditional method. Therefore, issues related to the preparation and transplantation of cells must be resolved so that cells with higher biological activities are obtained.

Cell sheet technology is a novel technology that is used in tissue engineering to prepare and transfer seed cells. In cell sheet technology, specific temperature-responsive polymers covalently bind to the surface of a Petri dish to provide a temperature-responsive Petri dish (1,2). The surface of the Petri dish is hydrophobic at 37°C, enabling cells to attach and proliferate. When the temperature is decreased to <32°C, the polymer surface is hydrophilic and a hydration layer is formed between the dish surface and the cells. Without using trypsin digestion, cells are automatically separated from the dish by cell sheet technology. Cells harvested by cell sheet technology contain extracellular matrix (ECM) and these ECM cells form a complete layer of the sheet structure with ion channels, growth factor receptors and connexins (3,4). Therefore, ECM cell layers made by cell sheet technology are more similar to normal tissue than the cells made by the traditional technology. Previously, cell sheet technology has been applied to construct various tissues and organs, including periodontal ligaments, skin, corneal epithelium and bladder epithelial tissue (5), as well as three-dimensional structures including cardiac muscle, smooth muscle, glomeruli and the hepatic lobule (6).

In the present study, canine bone mesenchymal stem cell (BMSC) sheets were prepared by cell sheet technology. In combination with canine demineralized bone matrix (DBM) and platelet-rich plasma (PRP), BMSC sheets were implanted into the latissimus dorsi of dogs and functional tissue-engineered bone was obtained. Immunoblot assays were performed to study the efficacy of the implantation by comparing the levels of the growth factors platelet-derived growth factor (PDGF) and vascular endothelial growth factor (VEGF) in the experimental group with those in a control group implanted with a DBM/PRP/BMSC complex.

## Materials and methods

### Animals and reagents

In total, 12 dogs weighing between 20 and 25 kg were provided by the Experimental Animal Center, Medical College Hospital of Qingdao University (Qingdao, China). DMEM low-glucose culture medium was purchased from Gibco-BRL (Carlsbad, CA, USA), the alkaline phosphatase kit was purchased from Nanjing Jiancheng Bioengineering Institute (Nanjing, China) and temperature reactive dishes were purchased from Nalge Nunc International (Tokyo, Japan). Femurs from the dogs were prepared by the Department of Oral and Maxillofacial Surgery. A scanning electron microscope (JEOL JSM-840) was purchased from Jeol Ltd. (Tokyo, Japan). All animal experiments were conducted according to the ethical guidelines of Qingdao University.

### Separation, culture and induction of BMSCs

Canine bone marrow (10 ml) was extracted and BMSCs were separated by gradient centrifugation (600 × g for 20 min) and placed in a flask at a density of 1×10^6^ cells/ml. Next, 10 ml low sugar DMEM culture medium was added and the flask was placed in a humidified incubator at a constant temperature of 37°C with 5% CO_2_ for culture and subculture. The second generation of BMSCs was inoculated in high-glucose DMEM osteogenic induction medium, containing 15% fetal bovine serum, 5 μg/ml vitamin C, 10 nM dexamethasone and 10 mM β-glycerophosphate.

### Alizarin red staining

Calcium nodules were stained with Alizarin red. Samples in the Petri dishes were washed twice with PBS, fixed with 95% ethanol for 10 min and then washed three times with water. The samples were stained with 0.1% Alizarin red-Tris-HCl (pH 8.3) at 37°C for 30 min. Samples were rinsed with distilled water, dried and then mounted for observation under an inverted light microscope (Olympus optical Co., Ltd., Tokyo, Japan).

### von Kossa staining

Samples in the Petri dishes were washed with PBS twice, fixed with 4% paraformaldehyde for 5 min and then washed three times with water. Next, 1 ml silver nitrate solution (5%) was added to the dishes and the samples were irradiated for 1 h under UV light. The silver nitrate solution was then removed and the samples were washed with distilled water three times. Next, 1 ml sodium thiosulfate solution (5%) was added to the dishes. After 1 min, the sodium thiosulfate solution was removed and the samples were dried at room temperature, followed by observation under a microscope.

### Preparation of the BMSC cell sheet

The fourth generation of BMSCs, with a density of 1×10^6^ cells/ml, was inoculated into a temperature-responsive dish with a diameter of 3.5 cm. The dish was placed in an incubator at 37°C with 5% CO_2_ and saturated humidity. When proliferative fusion of BMSCs reached 90%, the dish was placed in an incubator at 20°C with 5% CO_2_ and saturated humidity for 20 min. Following the shedding of the cell sheet, it was observed under an inverted microscope.

### Preparation of canine DBM

Soft tissue and periosteum were removed from the femurs of the dogs. The bones were preserved at −80°C for 6 months. Processes, including degreasing, decalcification and the removal of non-collagen protein, were performed. Canine allogeneic DBM with a size of 2×2×0.5 cm was formed following freeze-drying.

### Preparation and activation of PRP

PRP was prepared during surgery. In total, 30 ml blood was obtained from the femoral vein, using a sterile needle containing 4.2 ml anticoagulant. Following centrifugation at 4°C (160 × g for 20 min), a small number of red blood cells had precipitated at the bottom of the tube. The majority of the supernatant was discarded. The residual liquid (~1 ml) was the PRP, which was activated by the addition of 200 μl thrombin. The PRP was gently agitated for ~10 sec, until it formed a jelly, for wrapping the DBM/MSC complex.

### Construction of the BMSC/BMSC cell sheet and DBM/PRP complex

At 24 h prior to implantation, the BMSC suspension was slowly transferred on to the DBM until the DBM was completely soaked. Next, the samples were incubated at 37°C with 5% CO_2_ for 24 h. The PRP and the prepared cell sheet were gently placed on the surface of the DBM for implantation.

### Implantation

For anesthesia, 20 mg/kg ketamine was intramuscularly administered to the dogs. Back hair was removed bilaterally for skin preparation and disinfection. The skin and superficial fascia was dissected. When the flap was retracted, the edge of the latissimus dorsi was identified. The thoracodorsal artery and vein were dissected in the deep surface of the latissimus dorsi. The DBM/PRP/BMSCs/BMSC cell sheets were implanted in the left side and the DBM/PRP/BMSC complexes were implanted in the right side, to serve as a control in each dog. Penicillin (2,000,000 U/day) was intramuscularly injected every day for 1 week following surgery. The stitches were removed 14 days after the surgery. Two dogs were sacrificed at 4, 8 and 12 weeks following surgery, for gross observation and histological examination.

### Immunoblot assays

Total proteins were harvested from the tissues of the control and experimental implantation sites of the dogs. The proteins were separated on SDS-PAGE gels and subjected to immunoblot analyses. The primary antibodies anti-PDGF (sc-128; 1:200) anti-VEGF (sc-7269; 1:200) and anti-β-actin (sc-130301; 1:10,000) were purchased from Santa Cruz Biotechnology, Inc. (Santa Cruz, CA, USA). Secondary antibodies were horseradish-peroxidase-conjugated secondary anti-mouse IgG (31430; 1:10,000; Pierce Biotechnology, Inc., Rockford, IL, USA). Bound antibodies were detected using an electrochemiluminescence (ECL) system (Pierce Biotechnology, Inc.). Experiments were repeated >3 times. The developed film was scanned using the AlphaImager gel imaging systems (AlphaImager, Santa Clara, CA, USA). The scanned immunoblot images were analyzed using Quantity One software (Bio-Rad Laboratories, Hercules, CA, USA). β-actin was used as an internal control. The relative expression level of PDGF and VEGF was calculated based on the gray value of β-actin.

### Statistical analysis

SPSS 18.0 statistical software was used for statistical analysis (SPSS Inc., Chicago, IL, USA). Data were presented as mean ± standard deviation (SD). The difference between the control group and experimental group was analyzed using the Student’s t-test. P<0.05 was considered to indicate a statistically significant result.

## Results

### Cell morphology of the prepared BMSC cell sheet

**Figure 1:**
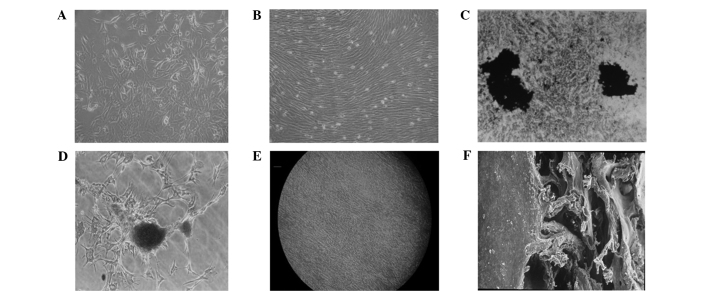
Cell morphology of the prepared BMSC cell sheet. (A) BMSCs following 7 days of primary culture (magnification, ×100). (B) BMSCs following 12 days of primary culture demonstrated whirlpool growth (magnification, ×100). (C) von Kossa staining showed two large black stained areas in a cell density zone (magnification, ×250). (D) Alizarin red staining of nodules (magnification, ×250). (E) Formation of the BMSC cell sheet, as observed under an inverted microscope, with cells in short spindle or pleomorphic shapes with unclearly defined boundaries (magnification, ×100). (F) The cell sheet was inoculated with good adhesion to the DBM, as observed under an electron microscope (magnification, ×100). BMSCs, bone mesenchymal stem cells; DBM, demineralized bone matrix.

During the preparation of the BMSC cell sheet, small numbers of primary cultured BMSCs were observed to be adherent within the first 24 h, which were in a polygonal, oval or pleomorphic growth state. At 72 h, the majority of cells were spreading and adherent. On day 7, visible colonies had formed, which grew in swirling or radial shapes (Fig. 1A). On day 12, cells were completely fused in a swirling shape, with the majority in long spindle shapes (Fig. 1B).

Following osteogenic induction, the proliferation of the BMSCs became significantly slower. On day 7, the shapes of the cells changed from long fusiforms to polygonal and square shapes. Between days 21 and 28, calcified nodules formed. The nodules were stained black by von Kossa staining and orange by Alizarin red staining. Black and white images of the staining are shown in Fig. 1C and D.

On day 7 after induction, the temperature-responsive dish containing the BMSCs was placed in an incubator at 20°C with 5% CO_2_. The cells gradually separated from the bottom of the dish. After 20 min, the cells and ECM were removed to form a complete cell sheet. Under a light microscope, the morphology of the cells was observed to have changed to a short spindle or pleomorphic shape, with unclearly defined boundaries (Fig. 1E). The cells gradually shrank to form a dense cell sheet detached from the bottom of the dish. One week following the inoculation of the cell sheet to the surface of the DBM, the DBM was wrapped by the cell sheet, as observed under a scanning electron microscope (Fig. 1F). These results indicate that at 20°C, BMSCs detached automatically from the temperature-responsive culture dishes to form an intact cell sheet.

### Histological analyses of osteoblasts

To determine the osteogenic effectiveness of the BMSC cell sheets, implantation into canine latissimus dorsi was performed. The dogs were implanted with the DBM/PRP/BMSC cell sheet/BMSC (the experimental group) and DBM/PRP/BMSC complexes (the control group). Stitches were removed from the implantation sites 14 days after surgery. Two dogs were euthanized at 4, 8 and 12 weeks following surgery for gross observation and histological examination.

**Figure 2:**
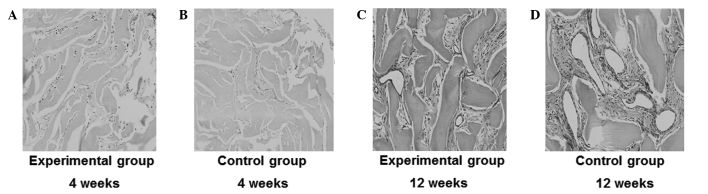
Histological analyses of osteoblasts using hematoxylin and eosin (magnification, ×100). (A) In the experimental group 4 weeks after implantation, active osteogenesis was observed with osteoblasts and numerous capillaries around the trabecular bone. (B) In the control group 4 weeks after implantation, a lower vascular density with trabecular fibrosis was observed. (C) In the experimental group 12 weeks after implantation, the trabecular bone was regular and thick with a high density. There was less fibrous tissue in the mature trabecular bone and the vascular density was decreased. (D) In the control group 12 weeks after implantation, there was less trabecular bone and there were numerous blood vessels with marked fibrosis.

Four weeks following implantation in the canine latissimus dorsi, active osteogenesis was observed in the experimental group, with considerable numbers of osteoblasts and capillaries detected around the trabecular bone (Fig. 2A). In the control group, the osteogenesis and vascular density were less evident, and trabecular bone fibrosis was visible (Fig. 2B). After 8 weeks, the trabecular bone was coarse, osteoblasts were active, capillaries were abundant between the trabeculae and fresh fibrosis was observed in the experimental group, whereas in the control group, the trabecular bone was coarse and the trabeculae had abundant vascularity and marked fibrosis (data not shown). After 12 weeks, the trabecular bone was regular and thick with a high density, fibrous tissue was present in the mature trabecular bone and the blood vessel density was reduced in the experimental group (Fig. 2C). In the control group, there was less trabecular bone and numerous blood vessels and marked fibrosis were observed (Fig. 2D). These results indicate that osteogenesis in the DBM/PRP/BMSC cell sheet/BMSC group was significantly more effective than that in the DBM/PRP/BMSCs group.

### PDGF and VEGF growth factor levels in the control and experimental groups

**Figure 3:**
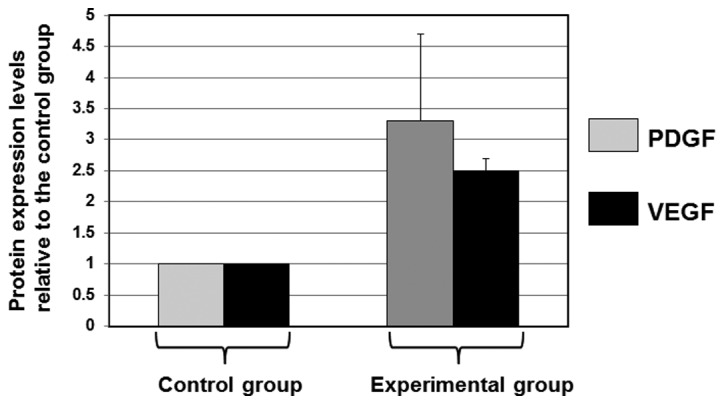
Immunoblot analyses of PDGF and VEGF growth factors in tissues from the control and experimental implantation sites of dogs. The bar chart shows the mean normalized OD of PDGF and VEGF protein bands relative to the OD of the β-actin band. The data are presented as mean ± SD (P<0.05).

To further determine the effectiveness of the BMSC cell sheets on implantation efficiency, total proteins were harvested from tissues in the control and experimental implantation sites of the dogs. The proteins were separated on SDS-PAGE gels and subjected to immunoblot analyses of the levels of the growth factors PDGF and VEGF. The cellular protein, β-actin, served as a loading control in the immunoblot analyses. The mean normalized OD of the protein bands, relative to the OD of the β-actin band, was calculated from each condition and subjected to statistical analysis. Error bars show the SD of the mean (Fig. 3). As shown in Fig. 3, the mean levels of PDGF and VEGF in the experimental group were 3.2- and 2.5-fold higher than the mean expression levels in the control group, respectively. These results indicate that BMSC cell sheets are functional and more effective than the control cell complexes.

## Discussion

In the present study, cell sheet technology was used to construct tissue-engineered bone. BMSCs were induced to differentiate into osteoblasts for BMSC cell sheet preparation. Using a scanning electron microscope, the prepared BMSCs were shown to be in a complete sheet structure, containing a layer of ECM. BMSC sheet layers retain cell surface proteins, including ion channels and connexin, which enable the effective transmission of signals and the maintenance of a coherent function (3). The BMSCs were arranged in a dense sheet layer, which was similar to natural bone during the formation of osteoblasts. The shrinkage-generated stress was due to a specific regulation effect on the polarization of BMSCs (7). A lamellar bone structure was formed that was similar to that of the surrounding mineral with regard to deposition and calcification. Single- or multi-layer BMSC-wrapped biodegradable scaffolds were implanted into the recipient area, which was expected to form a functional tissue-engineered bone having a lamellar bone structure comparable to that of normal bone.

The DBM prepared in the present study was constructed as a collagen grid with a consistent gap size. Scanning electron microscopy showed that the DBM had a three-dimensional mesh structure with a porosity of ~70%. Such three-dimensional mesh structures provide a surface area for cell adhesion and proliferation, which aids the nutrition, metabolism and angiogenesis of bone tissue. PRP is a platelet concentrate formed by the centrifugation of autologous whole blood. Following activation by thrombin and calcium chloride, the platelet α granules release PDGF, transforming growth factor (TGF)-β, VEGF, epidermal growth factor (EGF), insulin-like growth factor (IGF) and other growth factors (8–10). With the exception of IGF, the PRP concentrations of the remaining four types of growth factor were 3–8 times the concentrations in whole blood (11). In addition, PRP preparation is simple, fast, inexpensive and minimally invasive, and does not cause immune rejection or the spread of disease. A variety of growth factors in PRP are known to be important for the promotion of bone regeneration and angiogenesis (12).

In the present study, BMSCs were prepared by cell sheet technology, which avoided the use of trypsin digestion. By this method, cell damage was reduced and therefore, a considerable amount of ECM was retained, which greatly improved cell utilization and the biological activity of the transferred cells. Immunoblot assays were performed to study the effects of the two implant types by comparing the levels of growth factors PDGF and VEGF. The mean levels of PDGF and VEGF in the experimental group were 3.2- and 2.5-fold higher than the mean expression levels in the control group, respectively. The results obtained indicate that BMSC cell sheets are functional and more effective than the control cell complexes. Thus, cell sheet technology may be useful in constructing functional tissue-engineered bones.
